# Guillain-Barré syndrome with associated unilateral ptosis without ophthalmoplegia – a rare presentation: a case report and review of the literature

**DOI:** 10.1186/s13256-019-2157-x

**Published:** 2019-07-20

**Authors:** Udaya Ralapanawa, Prabhashini Kumarihamy, Thilak Jayalath, Jeevani Udupihille

**Affiliations:** 1 Department of Medicine, University of Peradeniya, Peradeniya, Sri Lanka; 2 Teaching Hospital, Peradeniya, Sri Lanka; 3 Department of Radiology, Teaching Hospital, Peradeniya, Sri Lanka

**Keywords:** Guillain-Barré syndrome, Isolated unilateral ptosis, Ophthalmoplegia, Cranial nerve enhancement, Magnetic resonance imaging

## Abstract

**Background:**

Guillain-Barré syndrome is an acute inflammatory polyradiculoneuropathy. Nearly half of patients with Guillain-Barré syndrome have cranial nerve involvement. However, isolated bilateral ptosis without ophthalmoplegia is a rare manifestation, and isolated unilateral ptosis without ophthalmoplegia in Guillain-Barré syndrome has not previously been reported in the literature. Furthermore, only few cases of Guillain-Barré syndrome with cranial nerve enhancement visualized by gadolinium-enhanced magnetic resonance imaging have previously been reported. We describe the first reported case of unilateral ptosis without ophthalmoplegia in Guillain-Barré syndrome and associated multiple cranial nerve enhancement seen by gadolinium-enhanced magnetic resonance imaging.

**Case presentation:**

Our patient was a 55-year-old Sinhalese man who was admitted to a tertiary care hospital in Sri Lanka with acute-onset progressive weakness in the lower limbs followed by the upper limbs. He had bilateral symmetrical flaccid quadriparesis with absent reflexes and flexor plantar response. Left-sided isolated partial ptosis without associated ophthalmoplegia was noted with normal pupils. The patient’s neurological examination was otherwise normal. A nerve conduction study showed a severe demyelinating type of polyneuropathy. No decremental response to repetitive nerve stimulation was observed, and the result of a single-muscle-fiber electromyogram was negative. A diagnosis of Guillain-Barré syndrome was made, and the patient was treated with intravenous immunoglobulin. His condition gradually deteriorated over the next few days, and he became quadriplegic despite the completion of immunoglobulin therapy. Later he developed multiple cranial nerve palsies, including bi-lateral lower motor neuron type facial nerve palsy, and he required mechanical ventilation. By this time, he had complete left-sided ptosis with a normal right eye. He never developed ophthalmoplegia or ataxia. Magnetic resonance imaging of the brain showed contrast enhancement in the intracranial part of multiple cranial nerve roots and basal leptomeninges. He gradually improved with plasmaparesis, and ptosis was the first to improve.

**Conclusions:**

Even though Guillain-Barré syndrome was recognized a century ago, there are still many unanswered questions about it and its florid presentation. Large-scale studies are needed for better understanding of its pathophysiology and prototypes and to find answers for still-unanswered questions. The clinician must have a high index of suspicion and be familiar with mimics and prototypes to diagnose Guillain-Barré syndrome accurately without delay.

## Background

Guillain-Barré syndrome (GBS) is an acute inflammatory polyradiculoneuropathy, and it is said to be immune-mediated. It is a heterogeneous disorder with several variants, and each variant has distinct features. Acute inflammatory demyelinating polyneuropathy is the most common variant. There are other prototypes with axonal neuropathies that are less common: acute motor axonal neuropathy, acute motor and sensory axonal neuropathy, and Miller Fisher syndrome (characterized by ophthalmoplegia, areflexia, and ataxia) [1, 2]. There are several other localized subtypes of GBS as well [2].

Nearly 50% of patients with GBS have cranial nerve involvement. Of all cranial nerves, the facial nerve is the most commonly affected, in the form of unilateral or bilateral involvement [3]. Ocular muscle involvement (occurring in nearly 10% of patients), and especially isolated ptosis without ophthalmoplegia, is a rare manifestation [3]. Even though there were several reported cases with isolated bilateral ptosis as an early sign of GBS [4–6], isolated unilateral ptosis without ophthalmoplegia in GBS has not previously been reported in the literature. Furthermore, there were few cases of GBS with cranial nerve enhancement visualized by gadolinium-enhanced magnetic resonance imaging (MRI) [7, 8]. We describe the first reported case of unilateral ptosis without ophthalmoplegia in GBS and associated multiple cranial nerve enhancement seen by gadolinium-enhanced MRI. When a patient develop ptosis with flaccid paralysis, it is very challenging to distinguish GBS from other mimics and variants of GBS, and the clinician should have a high index of suspicion, especially if the patient’s presentation is atypical.

## Case presentation

**Fig. 1:**
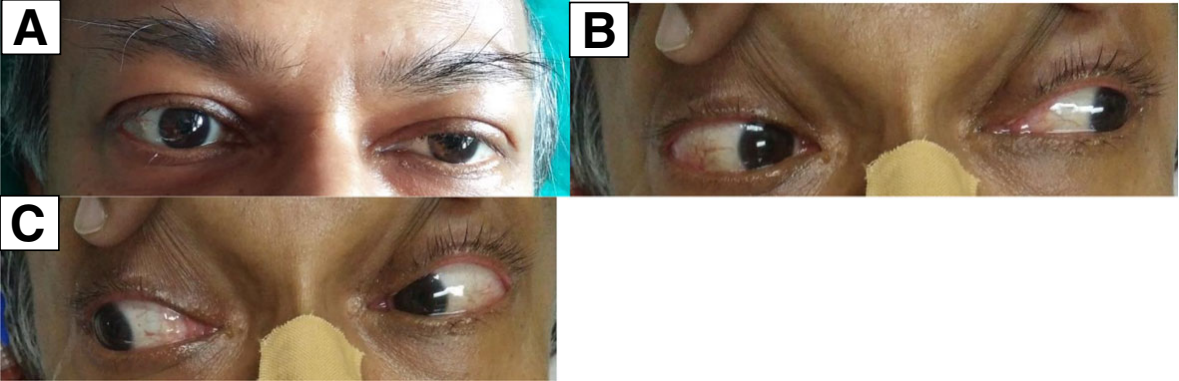
Clinical findings in the eyes. **a** Partial ptosis of left eye. **b** and **c** Absence of ophthalmoplegia

Our patient was a 55-year-old Sinhalese man who was admitted to a tertiary care hospital in central Sri Lanka with bilateral upper limb and lower limb numbness with associated weakness of 5 days’ duration. Five days earlier, he had felt numbness in his upper and lower limbs bilaterally and noticed weakness in his toes on the second day of illness. He had progressive weakness, and at the time of admission to the hospital, he had weakness in both upper and lower limbs. He had no associated respiratory difficulty, swallowing difficulty, or urinary or fecal incontinence or retention. He denied a history of preceding gastroenteritis and respiratory or other infections. Other than having hypertension, his past medical history was unremarkable. He denied alcohol abuse, smoking, or recent immunizations. On examination, his higher functions were normal, and he had bilateral symmetrical upper and lower limb weakness with prominent lower limb involvement. His distal muscles were weaker (grade 3) than his proximal muscles (grade 4). His limbs were flaccid, and all the reflexes were absent with flexor plantar response. He did not demonstrate any abnormalities in sensory, sphincteric, and coordination examinations. Left-sided isolated partial ptosis was noted without associated ophthalmoplegia (Fig. 1), and his pupils were of normal size, symmetric and reactive to light. He had no associated ataxia. He did not complain of any double vision and had no associated fatigability. His other cranial nerves were also normal. His cough reflex and neck muscle power were normal with 500 ml of spontaneous tidal volume. His blood pressure was 160/100 mmHg with a pulse rate of 72 beats/minute and a respiratory rate of 12 breaths/minute. The result of his full blood count was normal with normal erythrocyte sedimentation rate and C-reactive protein concentration. His liver and renal profiles were normal. His serum potassium, calcium, and magnesium levels were normal. A nerve conduction study showed a severe demyelinating type of polyneuropathy. No decremental response to repetitive nerve stimulation was observed, and the result of single-muscle-fiber electromyogram (EMG) was negative. The patient’s ptosis did not improve with the ice pack test.

A diagnosis of GBS was made, and the patient was treated in the high-dependency unit with supportive care and intravenous immunoglobulin (IVIg; a standard single IVIg dose [0.4 g/kg bodyweight/day] for 5 consecutive days). Lumbar puncture done on the tenth day of the patient’s illness showed cell protein dissociation (cerebrospinal fluid [CSF] protein was 670 mg/dl with no white cells). The results of Gram staining and CSF cultures were all negative. His condition was gradually deteriorated over the next few days, and he became quadriplegic despite completing 5 days of IVIg therapy. Later he developed bi-lateral lower motor neuron–type facial nerve palsy. Apart from these, he developed difficulty in swallowing and impaired palatal movement on the 15th day of his illness with no associated other cranial nerve involvement. By this time, his left-sided ptosis had progressed to complete ptosis with a normal right eye. He never developed ophthalmoplegia or ataxia. His neck muscle power became weak, and he required mechanical ventilation due to respiratory failure. MRI of the brain, cervical and thoracic spine showed an unremarkable brain, cerebellum, brainstem, and cervical and thoracic spine with contrast enhancement in the intracranial part of the optic nerve and the Vth, VIth, VIIth, and VIIIth cranial nerve roots in the right side with left VIIth and VIIIth cranial nerves and basal leptomeninges. No mass lesion or obstruction of CSF fluid was seen. Ten days after completion of IVIg therapy, plasma paresis was arranged because of worsening of the patient’s illness. Plasma exchange was started with an exchange of about 2 L of plasma every other day for 5 days. A gradual improvement of respiratory function and left-sided ptosis was observed without significant improvement in peripheral muscle strength after the second cycle of plasma exchange, and after the fifth cycle of plasma exchange, the patient was weaned from mechanical ventilation. The neurologist’s opinion was taken, and it was decided to complete ten cycles of plasmaparesis. The patient’s peripheral muscle strength started to improve gradually with the seventh cycle of plasma exchange, when his proximal muscles of both upper and lower limbs improved earlier than his distal muscles. By this time, the patient’s left-sided ptosis was totally improved. He was given physiotherapy and discharged from the hospital after nearly 2 months of illness. He was regularly followed up in the clinic, and his proximal muscle power in both upper and lower limbs was normal when he was examined 1 month after discharge. His distal muscles' power had only slight improvement even after 4 months of illness.

## Discussion

GBS is the most common cause of generalized acute flaccid paralysis seen in clinical practice. It has now been recognized as an increasingly diverse disease with several prototypes [1, 2]. Even though each variant has distinct features, the variants can present with atypical features, too. A study by Karimzadeh *et al.* that included 33 patients with GBS showed that 24.3% had atypical presentations [9]. When a patient presents with atypical features, it poses significant diagnostic challenges to the treating physician.

Once a patient presents with flaccid paralysis and ptosis, there are several other differential diagnoses that need to be considered. Miller Fisher syndrome, a variant of GBS, is one of them. It is characterized by ophthalmoplegia, areflexia, and ataxia [2]. Our patient never developed either ophthalmoplegia or ataxia during his illness. Furthermore, his nerve conduction studies showed a demyelinating pattern rather than an axonal pattern, which is seen in Miller Fisher syndrome [2]. CSF analysis typically reveals elevated protein concentrations without pleocytosis in GBS, as in our patient’s case [10]. The presence of protein cell dissociation in CSF is uncommon in Miller Fisher syndrome [11]. Patients with Miller Fisher syndrome show positive anti-GQ1b immunoglobulin G (IgG) antibodies [11]. In our patient, anti-GQ1b IgG antibodies were not done due to financial constrain. Stalpers *et al.* reported a case of a patient with the Miller Fisher variant of GBS who had isolated bilateral ptosis as the only ophthalmologic sign (without ophthalmoplegia), but their patient had ataxia and positive anti-GQ1b IgG antibodies [12].

Myasthenia gravis (MG) is a very important differential diagnosis we considered in our patient. However, it was excluded in our patient owing to the absence of fatigability or diurnal variation of symptoms, and further, no decremental response to repetitive nerve stimulation was observed, and the result of single-muscle-fiber EMG was negative. Because we did not have edrophonium, the Tensilon test was not done, and the patient’s ptosis did not improve with the ice pack test [13]. MG was excluded without a doubt in our patient. Botulism is another neuromuscular junction disorder that needs to be considered, and in our patient, it was excluded in the presence of normal pupillary function and in the absence of ophthalmoplegia. Also, descending paralysis is a feature of botulism rather than ascending paralysis, which was observed in our patient [14–16].

Normal MRI findings other than the contrast enhancement in the intracranial part of the optic nerve and the Vth, VIth, VIIth, and VIIIth cranial nerve roots in the right side with left-sided cranial nerves VII and VIII and basal leptomeninges excluded the possibility of brainstem diseases in our patient.

Hence, an atypical presentation of GBS was diagnosed in our patient in the presence of a characteristic severe demyelinating type of polyneuropathy based on electrophysiological study and protein cell dissociation in CSF analysis in association with acute ascending flaccid paralysis of the limbs with multiple cranial nerve involvement in the absence of any other explainable cause.

In approximately half of the patients with GBS, cranial nerves may be affected during the time course of the illness, and it may be preceded or followed by involvement of the extremities. Among cranial nerves, the facial nerve is the most commonly affected [3]. Only nearly 10% of patients may develop ocular involvement. However, isolated ptosis without ophthalmoplegia is a rare manifestation. Our extensive literature survey revealed several reported cases with isolated bilateral ptosis without ophthalmoplegia as an early sign of GBS [4–6]. In 1986, Ropper reported that of 92 consecutive patients with GBS, 8 had severe ptosis without ophthalmoplegia [6]. However, we could not find any case of isolated unilateral ptosis with normal pupils without ophthalmoplegia, which signifies the involvement of unilateral eyelid levators in association with GBS. Hence, our patient’s case seems to be the first such reported case.

Other than the facial and ocular muscle involvement, patients with GBS may have involvement of other cranial nerves, such as V, IX, X, and XI [2, 17, 18]. Our patient also had facial and bulbar muscles weakness, indicating involvement of multiple cranial nerves.

MRI of the brain and the cervical and thoracic spine in our patient showed contrast enhancement in the intracranial part of the optic nerve and the Vth, VIth, VIIth, and VIIIth cranial nerve roots in the right side with left VIIth and VIIIth cranial nerve roots and basal leptomeninges. There were few cases reported in the literature of GBS with cranial nerve enhancement seen by gadolinium-enhanced MRI [7, 8]. Hence, we report another case. The diseases like, malignancy and tuberculosis, which can cause enhancement of cranial nerves and leptomeninges, was excluded from the history, examination, and investigations in our patient. However, follow-up MRI performed to see the resolution was not obtained, owing to the unavailability of MRI facility freely. The underlying pathophysiological mechanism of abnormal enhancement of the cranial nerves in GBS is yet to be understood. Due to the surrounding inflammation, perineural structures get enlarged. This may result in local increase in gadolinium, thus resulting in enhancement on imaging [19]. In our patient, enhancement of bilateral VIIth cranial nerves correlated with the clinical finding of bilateral facial muscle weakness. Even though our patient had weakness in oropharyngeal musculature, we did not see enhancement in relevant cranial nerves. Conversely, the right optic nerve and cranial nerves V and VI as well as both VIIIth cranial nerves showed enhancement, but no clinical evidence of demyelination was found. Hence, large-scale studies in GBS are mandatory for proper understanding of the correlation of cranial nerve enhancement on MRI with clinical presentation and electrodiagnostic studies.

In nearly 60% of cases, a mild respiratory or gastrointestinal tract infection or immunization precedes the onset of symptoms by 1 to 3 weeks. However, almost every febrile infection has at one time or another been reported to precede GBS [20–22]. Nonetheless, we could not find any such association in our patient clinically.

There are two main effective treatment options other than the supportive care for patients with GBS: IVIg and plasma exchange. However, steroids are not beneficial as a treatment for GBS [23]. According to several large comparative studies, plasma exchange and IVIg were found to be equally effective in the management of GBS [24, 25]. However, in our patient, we had to use plasma exchange later because his condition deteriorated despite treatment with IVIg, and he had significant improvement with plasma exchange. Further comparative studies are needed to evaluate the efficacy of these treatment options and to see whether there are any differences in response with each variant of GBS.

## Conclusions

Although GBS was first recognized nearly a century ago, there are still numerous unanswered questions about GBS, its variants, and its florid presentation. Large-scale studies are needed for better understanding of its pathophysiology and prototypes and to find answers to still-unanswered questions. Atypical presentations of GBS such as in our patient’s case pose a significant challenge to the treating physician, and the clinician must have a high index of suspicion and be familiar with mimics and prototypes to diagnose GBS accurately without delay.

## Data Availability

Not applicable.

## Abbreviations

CSF: Cerebrospinal fluid
EMG: Electromyogram
GBS: Guillain-Barré syndrome
IgG: Immunoglobulin G
IVIg: Intravenous immunoglobulin
MG: Myasthenia gravis
MRI: Magnetic resonance imaging
